# Barriers to motivation for physical activity in patients with affective disorders

**DOI:** 10.1192/j.eurpsy.2025.1371

**Published:** 2025-08-26

**Authors:** E. Petrov, N. Semenova

**Affiliations:** 1 Pirogov Russian National Research Medical University; 2 Moscow Research Institute of Psychiatry, Moscow, Russian Federation

## Abstract

**Introduction:**

Affective disorders, including but not limited to major depressive disorder, bipolar disorder, and persistent depressive disorder, comprise a group of disorders characterized by clinically significant mood disturbances. Depression, which makes the most important contribution to the DALY index among all mental disorders, was the primary focus of this study. Physical activity, regardless of changes in body weight, has been shown to reduce symptoms of depression and the likelihood of a new episode of the disease.

**Objectives:**

This qualitative study aimed to explore the barriers to motivation for physical activity in patients with affective disorders.

**Methods:**

This study comprised a qualitative investigation using semi-structured interviews with thematic analysis. Following ethical approval, a convenience sample of 10 participants with affective disorders was drawn: all of the sample were female, aged 18 years or older, with 69% falling into the 27–35 years age bracket. Diagnostic and clinical information were collected, and barriers to engagement in physical activity were explored. All interviews were recorded and transcribed verbatim.

**Results:**

Ten face-to-face qualitative interviews were completed and lasted between 30 and 60 min. The findings were summarized under the key thematic areas of Anhedonia, Fatigue, Lack of time, Fear of condemnation, and Embarrassment, illustrated by texts. The key thematic areas were further grouped under the overarching themes of 1. Personal characteristics and the influence of low mood: anhedonia and fatigue; 2. External factors: need more time; 3. Social factors: embarrassment and fear of condemnation. Then, the following barriers were identified: “Lack of Strength” barrier, “Lack of Time” barrier, and “Rejection of physical characteristics” (or self-stigma) barrier.

**Conclusions:**

While small and exploratory, the study provides significant insights into the barriers to motivation for physical activity in patients with affective disorders. Although these findings are not generalizable to other populations or males with affective disorders, they offer valuable considerations for future research and interventions in this field. This study’s findings have profound implications for future psychosocial interventions for patients with affective disorders. By identifying and understanding the barriers to motivation for physical activity, it paves the way for more effective, individualized interventions, including those aimed at reducing self-stigma.

**Disclosure of Interest:**

None Declared

